# Polygenicity of Comorbid Depression in Multiple Sclerosis

**DOI:** 10.1212/WNL.0000000000207457

**Published:** 2023-08-01

**Authors:** Kaarina Kowalec, Kathryn C. Fitzgerald, Amber Salter, Casandra Dolovich, Arvid Harder, Charles N. Bernstein, James Bolton, Gary R. Cutter, Lesley A. Graff, Sara Hägg, Carol A. Hitchon, Yi Lu, Fred Lublin, Kyla A. McKay, Scott B. Patten, Amit Patki, Hemant K. Tiwari, Jerry S. Wolinsky, Ruth Ann Marrie

**Affiliations:** From the College of Pharmacy (K.K.), Rady Faculty of Health Sciences, University of Manitoba, Winnipeg, Canada; Department of Medical Epidemiology & Biostatistics (K.K., A.H., S.H., Y.L.), Karolinska Institutet, Stockholm, Sweden; Department of Neurology (K.C.F.), Johns Hopkins School of Medicine, Baltimore, MD; Department of Neurology (A.S.), UT Southwestern, Dallas, TX; Department of Internal Medicine (C.D., C.N.B., R.A.M.), Department of Psychiatry (J.B.), and Department of Community Health Sciences (J.B., R.A.M.), Max Rady College of Medicine, Rady Faculty of Health Sciences, University of Manitoba, Winnipeg, Canada; Department of Biostatistics (G.R.C., A.P., H.K.T.), University of Alabama at Birmingham; Department of Clinical Health Psychology (L.A.G.), and Department of Rheumatology (C.A.H.), Max Rady College of Medicine, Rady Faculty of Health Sciences, University of Manitoba, Winnipeg, Canada; Icahn School of Medicine at Mount Sinai (F.L.), New York, NY; Department of Clinical Neuroscience (K.A.M.), Karolinska Institutet, Stockholm, Sweden; Department of Community Health Sciences (S.B.P.), Cumming School of Medicine, University of Calgary, Alberta, Canada; and Department of Neurology (J.S.W.), McGovern Medical School, The University of Texas Health Science Center at Houston (UTHealth), Houston.

## Abstract

**Background and Objectives:**

Depression is common in multiple sclerosis (MS) and is associated with faster disability progression. The etiology of comorbid depression in MS remains poorly understood. Identification of individuals with a high risk of depression, through polygenic scores (PGS), may facilitate earlier identification. Previous genetic studies of depression considered depression as a primary disorder, not a comorbidity, and thus, findings may not generalize to MS. Body mass index (BMI) is a risk factor of both MS and depression, and its association may highlight differences in depression in MS. To improve the understanding of comorbid depression in MS, we will investigate PGS in people with MS, with the hypothesis that a higher depression PGS is associated with increased odds for comorbid depression in MS.

**Methods:**

Samples from 3 sources (Canada, UK Biobank, and the United States) were used. Individuals were grouped into cases (MS/comorbid depression) and compared with 3 control groups: MS/no depression, depression/no immune disease, and healthy persons. We used 3 depression definitions: lifetime clinical diagnoses, self-reported diagnoses, and depressive symptoms. The PGS were tested in association with depression using regression.

**Results:**

A total of 106,682 individuals of European genetic ancestry were used: Canada (n = 370; 213 with MS), UK Biobank (n = 105,734; 1,390 with MS), and the United States (n = 578 with MS). Meta-analyses revealed individuals with MS and depression had a higher depression PGS compared with both individuals with MS without depression (odds ratio range per SD 1.29–1.38, *p* < 0.05) and healthy controls (odds ratio range per SD 1.49–1.53, *p* < 0.025), regardless of the definition applied and when sex stratified. The BMI PGS was associated with depressive symptoms (*p* ≤ 0.001). The depression PGS did not differ between depression occurring as a comorbid condition with MS or as the primary condition (odds ratio range per SD 1.03–1.13, all *p* > 0.05).

**Discussion:**

A higher depression genetic burden was associated with approximately 30%–40% increased odds of depression in European genetic ancestry participants with MS compared with those without depression and was no different compared with those with depression and no comorbid immune disease. This study paves the way for further investigations into the possible use of PGS for assessing psychiatric disorder risk in MS and its application to non-European genetic ancestries.

Persons with multiple sclerosis (MS) are at a high risk of depression (incidence rate ratio 2.41, 95% CI 2.21–2.64).^1^ A retrospective cohort study of 2,312 people with MS found significantly greater annual disability progression, as measured by the Expanded Disability Status Scale (EDSS) over 10 years in those with a mood or anxiety disorder (β = 0.28, *p* = 0.0002).^2^ The effect of depression on mortality in MS is greater than the association of either MS or depression alone (attributable proportion 13%–14%).^3^ Despite the adverse effects of depression in MS, it remains underdiagnosed and undertreated.^4^ Therefore, an unmet need is the identification of individuals with a high risk of depression to potentially facilitate earlier screening or treatment.

The reasons for the co-occurrence of MS and depression are incompletely understood. Risk factors for depression in MS include increasing disability,^5^ disease course,^5^ and obesity.^6^ Another factor associated with a modulating risk of depression is genetic variation. Genetic variation can stratify individuals regarding disease risk, including for depression.^7^ Many common health conditions, including depression, are polygenic, meaning many genes underpin their pathophysiology. Genetic or polygenic scores (PGS) capture polygenicity and are the number of inherited common variants, weighted by their effects. PGS have been investigated in people with MS, most often as the MS disease risk PGS in connection with brain imaging outcomes,^8,9^ including a recent study that found modest associations between the MS PGS and changes in brain volume in European individuals with MS.^9^ Whereas the depression PGS was not associated with self-reported depression in 184 European individuals with MS with depression.^10^

Outside MS, a genome-wide meta-analysis of 246,363 cases with depression found individuals in the top depression PGS decile had 3.5-fold higher odds of depression (*p* = 1.68 × 10^−8^) compared with those in the first decile.^11^ This genome-wide association study (GWAS) of depression did not assess depression as a comorbidity, and thus, findings may not generalize to individuals of European genetic ancestry with other primary conditions such as MS. A separate GWAS of depression specifically in MS did not identify any significant variants associated with depression relative to MS, but the sample size included only 182 cases of depression.^12^

There are limited studies that have directly assessed whether the depression PGS is associated with depression in MS, especially using gold standard assessments of major depressive disorder (MDD). Thus, we aimed to determine whether the depression PGS is associated with comorbid depression in MS compared with the following: MS and no comorbid depression, depression not comorbid with an immune disease, and healthy controls. Given that obesity is a risk factor of MS and depression,^13^ we also considered PGS for body mass index (BMI). We sought to answer our research question using samples from Canada, the United Kingdom, and the United States to enhance precision and generalizability of our findings. We hypothesized that a higher depression PGS is associated with increased odds for comorbid depression in MS.

## Methods

### Study Design and Samples

We used samples from 3 existing studies from Canada, the United Kingdom, and the United States to generate a case-control study design.

#### Canada

The Canadian Institutes of Health Research Team on Defining the Burden and Managing the Effects of Psychiatric Comorbidity in Chronic Immunoinflammatory Disease is a prospective 3-year longitudinal study of immune-mediated inflammatory diseases.^14^ As previously reported, participants residing in Manitoba, Canada, were recruited between November 2014 and July 2016. Cohorts included participants with MS, a lifetime history of depression disorders but no chronic immune disease, or healthy controls. Multiple recruitment methods were used including poster placement in hospitals, private medical clinics, and educational institutions. For the MS cohort, recruitment included in-person or telephone calls or mailouts to patients from community-based and tertiary care clinics. Blood samples were collected in addition to sociodemographic and clinical data. Participants were required to be aged 18 years or older and had to be sufficiently proficient in English to complete questionnaires.

#### The United Kingdom

The UK Biobank (UKB) is a population-based cohort of >500,000 individuals aged 37–73 years from the UK who were recruited in the period 2006–2010.^15^ Participants were invited to answer touchscreen questionnaires at an assessment center, which contained sections about diseases, followed by a research nurse–led interview for further details regarding the diseases reported. In addition, linked hospital admission records using *International Classification of Diseases, Tenth Revision* (ICD-10) codes were used to identify diagnoses from hospitals. Of the original cohort, approximately 158,000 individuals completed a web-based mental health questionnaire, which included the Composite International Diagnostic Interview– Short-Form (CIDI-SF).^16^ Blood samples were collected. From this cohort, we selected individuals who had MS, had a lifetime history of depression but no immune disease, or healthy controls.

#### The United States

The CombiRx trial (ClinicalTrial.gov: NCT00211887) was a 3-arm, randomized, multicenter, Phase 3 trial of combination MS disease-modifying therapies (interferon-β1a and glatiramer acetate vs either agent alone).^17^ Criteria for the study included neurologist-confirmed MS, with ≥2 relapses in the prior 3 years; aged 18–60 years, EDSS = 0–6, and no relapses in the 30 days before screening and randomization. Those completing the 3-year core study were invited to the extension, which included an additional 4 years (a total of 7 years of follow-up). Blood samples were collected. The US samples were used only for comparing comorbid depression in MS with MS without depression, given this was a MS clinical trial sample.

### Participant Definitions and Measures

We used 4 groups of participants: (1) MS and depression (cases), (2) MS and no depression (control), (3) depression and no immune disease (control), and (4) healthy (control).

To increase sample size, which is of importance in genetic studies, we included data from 3 different studies. When retrospectively combining data from multiple sources, a common issue is the phenotypes were differently assessed. We harmonized the depression phenotypes between the studies, which included comparing a gold standard depression measure (Structured Clinical Interview for the Diagnostic and Statistical Manual of Mental Disorders [SCID-DSM-IV]) in the Canadian sample with that of a comparable version in the UKB sample, which combined *ICD-10* diagnoses and the CIDI-SF. We then expanded this definition to include a self-reported measure of depression and another related, but different construct: depressive symptoms (Patient Health Questionnaire–9 [PHQ-9]). We had 1 measure of depression in the US sample, which was self-reported depression.

#### Canada

Cases were defined as those with neurologist-confirmed MS and depression. Depression was defined in 3 ways: (1) lifetime depression was assessed using the gold standard SCID-DSM-IV,^18^ (2) self-reported depression diagnosed by a doctor, which was derived from a questionnaire validated for use in MS,^19^ and (3) baseline PHQ-9, a validated 9-item self-reported tool that assesses depressive symptom severity over the previous 2 weeks, with a total score of 0–27.^20^ We used a validated PHQ-9 cutoff for the presence of at least moderate depressive symptoms (PHQ-9 ≥10).^20^

Three control groups were defined: persons with MS and no comorbid depression; depression without a comorbid immune disease, and healthy controls. Depression not comorbid with an immune disease was defined the same as for depression in individuals with MS. Immune diseases that were excluded from this group are summarized in eTable 1, links.lww.com/WNL/C885). Healthy controls were excluded for the following reasons: any chronic medical condition (eTable 1), known cognitive impairment, any positive response to the SCID-DSM-IV screening questions for depressive/anxiety disorders, any head injury associated with loss of consciousness or amnesia, or chronic medication use with the exceptions (contraceptives, hormone replacement therapy, transient antibiotic use, or multivitamins).

Self-reported questionnaires captured the following at baseline: sex, date of birth, annual household income, highest level of education attained (high school or lower: elementary school, junior high school, high school diploma/general educational development vs above high school: college, technical/trade, university), years of education, and smoking history. To facilitate harmonization, we categorized the following: annual household income (Canadian dollars: <$50,000, ≥$50,000, or “declined”), educational attainment (high school or below, above high school), and ever-smokers (lifetime smoked ≥100 cigarettes).^14^ A research assistant measured weight and height for BMI (kg/m^2^).

#### The United Kingdom

We defined people with MS using ≥2 conditions: primary or secondary hospital admission code (*ICD-10*: G35), lifetime baseline self-reported condition (data field 20002: 1261), or lifetime self-reported MS disease-modifying therapy (any of the following: interferon-β/interferon β-1b 1a/avonex/betaferon, and glatiramer/copaxone).^21^ Newer MS disease-modifying therapies were not included given the recruitment period predated their availability.

Similar to the Canadian sample, cases were defined as persons with MS and comorbid depression. We defined lifetime depression using the presence of either primary or secondary hospital admission codes for depression (*ICD-10*: F32/F33) or CIDI-SF–based MDD.^16^ We also included lifetime baseline self-reported condition (data field 20002: 1286) and a baseline PHQ-9 measure, which was also dichotomized to define moderate depressive symptoms (PHQ-9 ≥10). Three control groups were defined as follows: persons with MS and no comorbid depression; depression not comorbid with an immune disease, and healthy controls. Depression that was not comorbid with an immune disease was defined in the same manner as depression in those with MS (*ICD-10*/CIDI-SF, self-report depression or using the PHQ-9), and immune diseases were excluded (eTable 1, links.lww.com/WNL/C885). Healthy controls were defined similar to the Canadian sample by excluding chronic conditions using hospital admission codes or self-reported conditions (eTable 1).

Self-reported questionnaires captured the following at baseline: sex, age, BMI, annual household income (converted from 1 British Pounds to 1.61 Canadian dollars and into: <$50,000, ≥$50,000, or “declined”), highest education achieved ([high school or lower]: A levels/AS levels or equivalent, Certificate of Secondary Education or equivalent, O levels/General Certificate of Secondary Education or equivalent vs [above high school]: National Vocational Qualification or Higher National Diploma or higher National Certificate or equivalent, College or University degree, Other professional qualifications e.g., nursing, teaching vs [other]), and years of education. Ever-smokers were defined as current or ever tobacco smoking on most days or occasionally.

#### The United States

Participants had neurologist-confirmed MS. Depression comorbidity was assessed in a self-reported manner using medical history (at trial enrollment) and the use of concomitant medications (enrollment and follow-up). The following were captured at the clinical trial enrolment visit: sex, age, BMI, highest level of education attained (high school/above high school), and smoking history (ever/never).

### Genotyping, Quality Control, and Imputation

Further details of the genotyping, quality control, and imputation can be found in the eMethods. Genotype data were available on 445 Canadian and 599 USA participants. There was genotype data available for 487,410 UK participants following central quality control and imputation. To determine genetic ancestry, we performed principal component analysis in PLINK using the 1000 Genomes phase 3 v5 data as the reference (N = 2,493 unrelated individuals, by “Superpopulation”: 659 African, 347 Admixed, 504 East Asian, 503 Europeans, 480 South Asian) along with our study data.^22^ We excluded any samples that were further than 3 SDs from the 1000 Genomes European superpopulation reference on principal components 1 or 2 (N removed: 75 Canada; 27,700 UKB; 21 United States).^22^ After removing the non-European genetic ancestry participants, the principal components were regenerated without the reference data and were used as covariates.

### PGS Generation

Details are available in the eMethods (eTable 2, links.lww.com/WNL/C885), but briefly, PGSs were calculated using summary statistics from recent GWAS for depression^11,23^ and BMI.^24^ PGS were generated as the sum of the risk allele scores, weighted by their effects from the discovery GWAS and standardized to a mean of 0 (SD = 1).

### Statistical Analyses

We described the 3 samples regarding their characteristics using either the median (interquartile range), mean (SD) or frequency (%). To test whether PGS was associated with depression or depressive symptoms in MS, we used multivariable logistic regression (binary outcome) or linear regression (continuous outcome for PHQ-9 scores). We reported the results as odds ratios (ORs) for logistic models or β estimate (linear models) per 1-SD increase in PGS. We included the following covariates into the models: age (continuous), sex, and first 5 genetic ancestry principal components. We compared those with MS-comorbid depression (case) with: (1) MS-no comorbid depression, (2) depression-no comorbid immune disease, and (3) healthy controls in each sample. The summary results for each comparison were then meta-analyzed across samples using a fixed-effect inverse variance–weighted model, when between-study heterogeneity (*I*^2^) was ≤50%, or random-effect (*I*^2^ > 50%) inverse variance–weighted model.^25^ Given the association of BMI with MS and depression, we also considered baseline BMI in place of PGS in our analyses comparing MS-depression with MS-no depression and depression-no immune disease. Last, given male individuals with MS experience higher relative rates of depression than female individuals with MS,^26^ we performed sex-stratified analyses comparing MS-depression (case) with MS-no comorbid depression.

Statistical significance level was set at *p* ≤ 0.05. We did not impute missing data (other than the genetic data). Analyses were performed using R (v.4.1.2) with the following packages: *tidyverse*, *data.table*, *metafor*, and *cowplot*.

### Standard Protocol Approvals, Registrations, and Patient Consents

The Canadian study was approved by the University of Manitoba Health Research Ethics Board and Shared Health/Winnipeg Regional Health Authority, and all patients provided informed consent. Participants in the US study provided informed consent, and ethical approval was granted by the respective collecting institution. The UKB had obtained informed consent from all participants included in this study, with the study originally approved from the Karolinska Institutet Ethics Committee.

### Data Availability

Individual participant data collected for the US samples cannot be shared, but access may be granted through authorization from the Executive Committee. The Canadian dataset cannot be shared because some participants did not agree to data sharing. An application to access UK data is available through the UKB. Analyses code is available at: github.com/kkowalec/ms-depression.

## Results

We included a total of 106,682 European genetic ancestry samples from 3 countries or sources: Canada, n = 370 (213 with MS, 57%); UKB, n = 105,734 (1,390 with MS, 0.8%); and the United States, n = 578 (100% MS) (Table 1). Persons with MS from the US sample were younger and reported lower rates of smoking, compared with persons with MS in the Canadian and UKB samples (Table 1). The UKB sample is a population-based study and as such had lower rates of depression, compared with the Canadian and US samples, with the rates of comorbid depression in people with MS, depending on the definition, ranging from 40.8% to 45.1% (Canada), 14.7% to 16.3% (UKB), and 56.8% (the United States) (data not shown). The rate of self-reported depression was similar between the 2 samples collected from MS clinics and clinical trial settings (Canada: 45.1%; the United States: 56.8%). The overlap between the 3 measures of depression varied, with 30.5% (Canada) participants with MS having comorbid depression as defined by all 3 measures (eFigure 1, links.lww.com/WNL/C885), whereas 5.7% of the UKB MS population had depression as defined by all definitions.

**Table 1:**
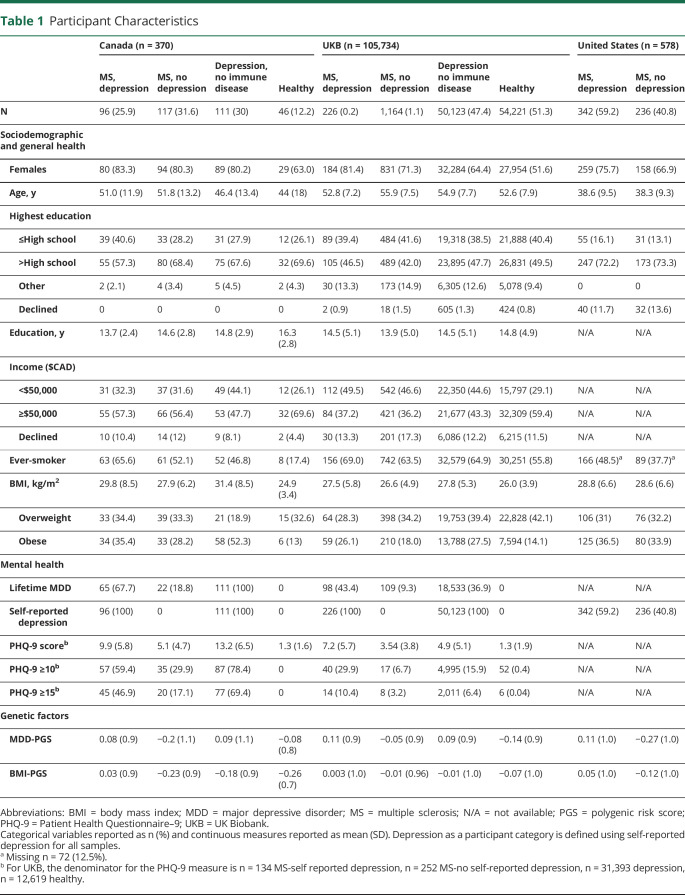
Participant Characteristics

**Figure 1:**
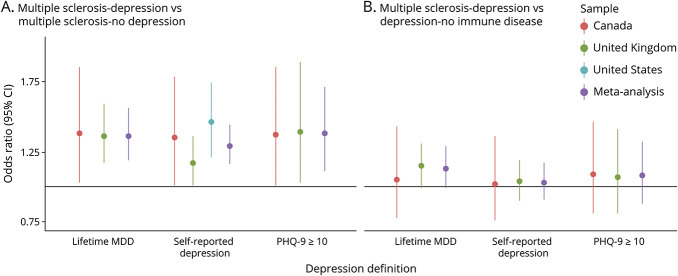
Multivariable Logistic Regression Analyses Investigating the Association Between the Depression Polygenic Score With MS and Comorbid Depression Compared With (A) MS and No Depression or (B) Depression With No Comorbid Immune Disease Each depression definition is assessed as a separate model including the polygenic scores for depression and BMI, the first 5 genetic ancestry principal components, age, and sex. BMI polygenic score results are reported in Table 3. Data represented as odds ratio and 95% CI. Random-effect inverse variance-weighted model was used for self-reported depression (heterogeneity A: *I*^2^ = 41.0%, B: *I*^2^ = 0%), whereas the other meta-analytic results used a fixed-effect model (heterogeneity lifetime MDD A: *I*^2^ = 0%, B: *I*^2^ = 38.5%; PHQ-9 ≥10 A: *I*^2^ = 0%; B: *I*^2^ = 43.2%). This figure visualizes the content of eTable 3 (links.lww.com/WNL/C885). BMI = body mass index; MDD = major depressive disorder; MS = multiple sclerosis; PHQ-9 = Patient Health Questionnaire-9.

Using any of the 3 depression definitions, meta-analyses revealed persons with MS and comorbid depression had a significantly higher depression genetic burden compared with both individuals with MS-no depression (OR per 1-SD increase in PGS 1.29–1.38, Figure 1, eTables 3 and 4, links.lww.com/WNL/C885) and healthy controls (OR per 1-SD increase in PGS 1.49–1.53, Table 2). The effect sizes were similar regardless of the measure used to define depression. Genetic variation associated with BMI was not significantly associated with MS-depression compared with either MS-no depression nor to healthy controls (Table 3). In the Canadian sample, the BMI PGS was significantly associated with self-reported depression and moderate depressive symptoms in MS (Table 3). Given the lack of any association between the BMI PGS and comorbid depression in the meta-analyses, to ensure BMI PGS was associated with measured BMI in our samples, we did find a strong correlation between BMI and BMI PGS in all samples used (eTable 5). We repeated the primary analyses using baseline BMI, instead of BMI PGS, and again found no overall effect in association with depression in MS (eTables 6 and 7).

**Table 2:**
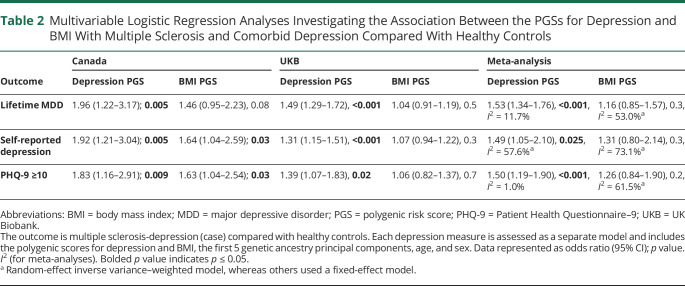
Multivariable Logistic Regression Analyses Investigating the Association Between the PGSs for Depression and BMI With Multiple Sclerosis and Comorbid Depression Compared With Healthy Controls

**Table 3:**
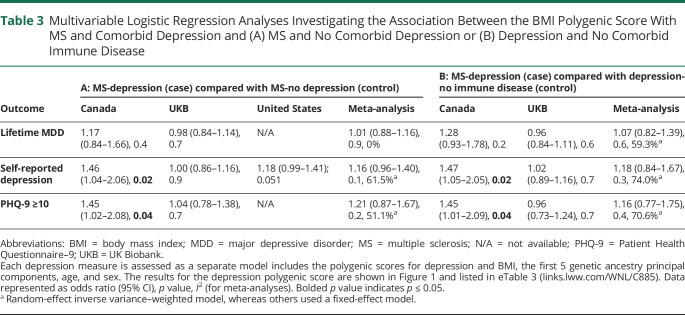
Multivariable Logistic Regression Analyses Investigating the Association Between the BMI Polygenic Score With MS and Comorbid Depression and (A) MS and No Comorbid Depression or (B) Depression and No Comorbid Immune Disease

We then compared the depression PGS between MS-depression with depression not comorbid with an immune disease. The meta-analyses showed the depression genetic burden in MS with comorbid depression was not significantly different from that of depression without a comorbid immune disease (Figure 1). The BMI PGS was also not significantly associated with MS-depression compared with depression without a comorbid immune disease (Table 3), apart from self-reported depression and moderate depressive symptoms in the Canadian sample (Table 3).

We used a continuous outcome (PHQ-9) to define current depressive symptoms in the Canadian and UKB samples (eTable 8, links.lww.com/WNL/C885). In the meta-analysis, a higher cumulative genetic burden for depression and BMI were associated with current depressive symptoms (depression PGS β = 0.28, BMI PGS β = 0.13, both *p* ≤ 0.001, eTable 8). We stratified these analyses by BMI category and sex, with the meta-analytic effects and standard errors in a similar size and direction to those of the unstratified meta-analysis.

We stratified the MS-depression compared with MS-no depression analyses by sex (Table 4, eTables 9 and 10, links.lww.com/WNL/C885). We observed similar proportions of male and female individuals in each sample by depression measure (eTable 9). The stratified meta-analyses revealed similar results to those of the unstratified (Table 4). The BMI PGS was not associated with any depression definition in the sex-stratified meta-analysis models (eTable 10).

**Table 4:**
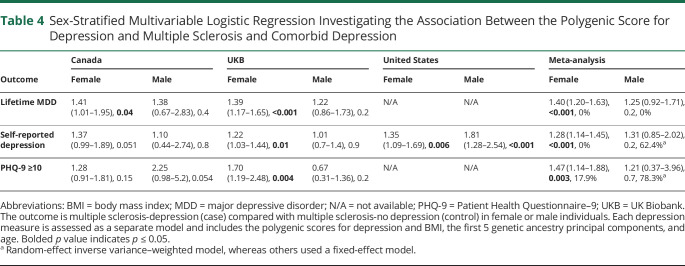
Sex-Stratified Multivariable Logistic Regression Investigating the Association Between the Polygenic Score for Depression and Multiple Sclerosis and Comorbid Depression

## Discussion

We used a large sample of approximately 106,000 individuals, predominantly from the UK, including 2,181 people with MS, from 3 countries to confirm our hypothesis that the depression PGS is associated with comorbid depression in MS. We found that a 1-SD increase in the depression PGS is associated with approximately 30%–40% increased odds for depression in persons with MS. We also found the PGS for depression had a similar association with depression among individuals with MS and in those without a comorbid immune disease.

In this study, we found that depression polygenicity plays a role in comorbid depression in MS and did not change based on sex nor when compared with depression occurring as the primary disease. A similar increase in the hazard (approximately 30%) for hospital-based depression diagnosis was identified in a Danish study of 34,573 individuals from the general population,^27^ when depression occurred as a comorbidity of Alzheimer disease,^28^ and to that of approximately 7,000 moderate-to-severe cases with MDD.^23^ Our study also provides evidence of a biological basis of depression for people with MS, whereas previous studies have identified conflicting associations between a family history of depression and the occurrence of depression in MS.^29,30^ This study uses a direct measure of genetic variation of depression, as opposed to family history, which captures genetic and environmental influences. Further understanding of the biological basis of depression in people with MS could be uncovered by either candidate gene studies^31^ or by a GWAS of depression specifically in those with MS. However, candidate gene studies in depression, in general, have not largely been supported,^32^ and a GWAS of depression including < 200 participants with depression and MS yielded no genome-wide significant findings.^12^

The effect size of the association between depression PGS and comorbid depression in MS from our study did not largely differ based on whether depression was assessed either as a lifetime or current condition or using ICD diagnostic codes, psychiatric interview, or depressive symptoms. The most recent large-scale GWAS of depression, which was used in this study to compute the PGS in the Canada and US cohorts, included >240,000 cases of depression collected from a variety of sources, including those ascertained from clinics and the broader group of self-declared affected individuals.^11^ A study including 12,106 individuals with MDD found similarly that the effect of the depression PGS did not vary depending on the depression definition.^33^ A study specifically in MS also found that the same depression PGS used in this study was not associated with the development of self-reported depression (hazard ratio 1.04, 95% CI 0.99–1.08, *p* = 0.063), but included a much smaller number of cases with depression (N = 184, 20.1%).^10^

BMI is a known risk factor for the development of both MS^13^ and depression.^34^ We found that BMI polygenicity was not significantly associated with comorbid depression in MS, compared with any of the control groups, nor in the sex-stratified models, potentially due to the low variance explained by the BMI PGS. However, our meta-analyses showed that a 1-SD increase in the BMI PGS was associated with increasing depressive symptoms (as measured by the PHQ-9) and when stratified by BMI category. Outside the field of MS, a Generation Scotland study including 13,921 individuals found that the BMI PGS was not associated with a lifetime SCID diagnosis of depression, although it was associated with increasing psychological distress.^35^ BMI-increasing genetic variation might be associated with increasing depressive symptoms but not a clinical lifetime diagnosis of depression in MS. We previously demonstrated in those with an immune disease, including MS, rheumatoid arthritis, or inflammatory bowel disease, that a 1-SD increase in the BMI genetic burden had 2.31 greater odds of high depressive symptoms, whereas those without an immune disease did not show this association.^36^ Along with this study, our results collectively point to possible differences in depressive symptoms compared with meeting a disorder definition. In addition, clinical subtypes of depression, such as atypical depression, are known to be differentially associated with BMI genetic variation,^37^ and future studies including subtypes of depression in MS may help with addressing this area further.

Regarding understanding the development of depression in MS, large administrative health data studies have shown that the rate of depression is elevated in the 5 years before MS diagnosis^38^ but Mendelian randomization studies have shown that depression is not causally linked to MS.^13^ The reason for their co-occurrence might therefore be related to chance, improved detection due to increased health service use, or genetic or environmental risks. Obesity is a shared risk factor between depression and MS, which, in this study, we found to be associated with depressive symptoms in MS, although other common risk factors such as smoking may also be explored in future work. The etiology of depression in MS may be heterogeneous. In some individuals with MS, depression may also occur secondary to structural changes in the brain.^39^

Our findings may have clinical relevance for people with newly diagnosed MS because they could be screened for their risk of depression using the depression PGS and offered counselling or pharmacologic therapies early, as preventative medicine. Preventative strategies, such as cognitive behavioral and problem-solving therapy, for depression to reduce its burden has shown success in targeted groups, including in newly diagnosed epilepsy.^40^ Screening for depression using PGS has not yet been effective, but in subgroups, for example, in people with MS, where the base rate of depression is higher than in the general population, theoretically, depression screening using PGS may prove useful. It could also allow health care to be more proactive, for example, harmless interventions such as mental health literacy training might be appropriate for a high-risk group (i.e., with a high depression PGS) who have not yet become depressed. This might also be a better way to encourage earlier intervention than formal screening. The identification of depression in MS is often complicated by the presence of somatic symptoms (e.g., fatigue, difficulty sleeping) that are characteristic of both depression and MS. Additional tools for the identification of depression, such as the depression PGS, may prove useful in these scenarios.

The strengths of this study include a large sample size, inclusion of different countries, and multiple definitions of depression. This provides replication and evidence of generalizability regarding the depression PGS and its application to European cohorts of people with MS. Although our study had multiple definitions of depression, we did not have depression subtype information, which may have proven helpful in identifying heterogeneity between depression occurring as a comorbid condition rather than the primary condition.^41^ The inclusion of the UKB as a sample in our study represents a strength due to its comprehensiveness and size. However, the UKB is also limited by a “healthy volunteer” selection bias,^42^ whereby individuals who volunteered for the UKB are more health conscious than nonparticipants. This likely led to the lower rates of depression in the UKB sample compared with our clinical samples from Canada and the United States. In this study, we did not aim to generate prevalence or incidence rates of depression but rather examine the association between a genetic exposure (depression PGS) with depression, and we were able to generate widely generalizable results between 3 international samples. Including additional measures of external validity, such as external samples or additional phenotype measures, along with the UKB has been noted to be important when performing research studies using UKB data.^43^ Some findings did not replicate between samples, including a significant association between BMI PGS with either self-reported depression or moderate depressive symptoms in MS, and may require a larger sample from individuals with MS. Future areas of research to further unravel the heterogeneity of depression in MS could include information on the treatment of depression and clinical features or depression subtype information.

Another limitation was that our findings are only generalizable to those of European genetic ancestry. The decision to select individuals only of European genetic ancestry is supported by empirical evidence, including differences in allele frequencies, linkage disequilibrium, and causal effect sizes between populations, which results in risk prediction of diseases to vary by population.^44,45^ A recent GWAS of depression in those of East Asian genetic ancestry found none of the European genetic ancestry depression loci to be significant,^46^ with similar findings in an African American study.^47^ Subsequently, applying the depression GWAS results from a European population to a non-European population would result in low transferability and a reduction in the PGS predictive power.^44^ There are methods available for computing transancestry PGS (e.g., PRS-CSx), but our study had a limited number of non-European genetic ancestry samples (e.g., East Asian or African) to apply these methods. Collectively, this highlights the need for future collection of samples to include more diverse populations with extensive data collection (e.g., to identify depression and MS) and may include the Million Veterans Program or the *All of Us* Research Program. However, each of these biobanks would individually have small numbers of people with MS; thus, specific targeted collection may be necessary. It is also important to further develop other aspects of the “genetic discovery pipeline” including biobanks with coverage on entire populations (e.g., using samples from newborn screening programs as is performed in Australia^48^), genotyping arrays, and imputation panels with better multiancestry coverage. Aside from genetic studies, underrepresentation is often prevalent across MS research, including a lack of diversity in clinical trials^49^ and other research studies.^50^ This could be improved by implementing a multifaceted approach to address barriers such as healthcare distrust, investing in the enrolment in underrepresented regions, and using inclusivity tools such as Dynamic Consents. Additional recommendations are available in the eAppendix 1 (links.lww.com/WNL/C885).

We found that a 1-SD increase in the depression genetic burden was associated with approximately 30%–40% increased odds of depression in European genetic ancestry participants with MS, irrespective of the depression definition, compared with both individuals with MS with no comorbid depression and healthy controls. Individuals with MS and comorbid depression had a similar genetic burden of depression compared with those with depression and no comorbid immune disease. Future studies replicating this association in a large cohort of non-European genetic ancestry is imperative. In conclusion, although PGS are not currently used in clinic for assessing the risk of depression, our findings indicate that future studies are warranted to determine whether depression PGS testing in subgroups, such as people with MS, may be more appropriate when the base rate of depression is higher, allowing more precise approaches to the management of depression to those groups.

## Glossary

BMI: body mass index
CIDI-SF: Composite International Diagnostic Interview-Short Form
EDSS: Expanded Disability Status Scale
GWAS: genome-wide association study
*ICD-10*: *International Classification of Diseases, Tenth Revision*
MDD: major depressive disorder
MS: multiple sclerosis
OR: odds ratio
PGS: polygenic score
PHQ-9: Patient Health Questionnaire–9
SCID-DSM-IV: Structured Clinical Interview for the Diagnostic and Statistical Manual of Mental Disorders
UKB: UK Biobank
